# Synthesis and Cytotoxic Evaluation of Novel *N-*Methyl-4-phenoxypicolinamide Derivatives

**DOI:** 10.3390/molecules16065130

**Published:** 2011-06-20

**Authors:** Wei Li, Xin Zhai, Lu Ding, Limin Sun, Xiaomei Chen, Ping Gong, Tiemin Sun

**Affiliations:** Key Laboratary of Original New Drugs Design and Discovery of Ministry of Education, School of Pharmaceutical Engineering, Shenyang Pharmaceutical University, Shenyang 110016, Liaoning, China

**Keywords:** *N-*methyl-4-phenoxypicolimamide derivatives, synthesis, cytotoxic activity

## Abstract

A series of *N-*methyl-4-phenoxypicolinamide derivatives were synthesized and evaluated *in vitro* for their cytotoxic activity against A549, H460 and HT29 cell lines. Pharmacological data indicated that some of the target compounds possessed marked antiproliferative activity, superior to that of the reference drug sorafenib. As the most promising compound, **8e** exhibited potent cytotoxicity with the IC_50_ value of 3.6, 1.7 and 3.0 μM against A549, H460 and HT-29 cell lines, respectively.

## 1. Introduction

Although great progress has been achieved in the treatment of cancer, it is still the leading cause of death, therefore the discovery and development of chemotherapeutics with novel structure and mechanism has been the challenge of medicinal chemistry. Sorafenib, a novel oral multiple-targeted antitumor drug with a diarylurea skeleton, has been approved by the FDA for the treatment of primary renal carcinoma and primary liver cancer [1,2,3], and research on potent sorafenib analogs has been the focus of many studies [4,5,6,7,8,9]. Interestingly, recent optimizations of the diarylurea framework of sorafenib led to discovery of benzimidazole- and benzioxazole-based sorafenib analogs with excellent antitumor activity, and the *N*-methyl-4-phenoxypicolinamide motif was retained in both studies to be the binding element of the hinge region which differs from the previous modifications [4,5].

Inspired by the recent paradigm of rational anticancer drug design [10,11] and the experience of reported modifications of the diarylurea backbone, a series of novel *N*-methyl-4-phenoxypicolinamides were designed by hybridizing the *N*-methyl-4-phenoxypicolinamide motif with either 5-aryl-1,3,4-thiadiazol-2-ylamino or 4-arylthiazol-2-ylamino groups in continuation of our interest in modifications on sorafenib. In this paper, we report the synthesis and cytotoxicity of *N*-methyl-4-(4-(5-aryl-1,3,4-thiadiazol-2-ylamino)phenoxy)picolinamides **8a–8k** and *N*-methyl-4-(4-(4-arylthiazol-2-ylamino)-phenoxy)-picolinamides **10a–10e**. Preliminary structure-activity relationships (SARs) were discussed to provide guidance for further study of *N-*methyl-4-phenoxypicolinamide derivatives.

## 2. Results and Discussion

### 2.1. Chemistry

**Scheme 1:**
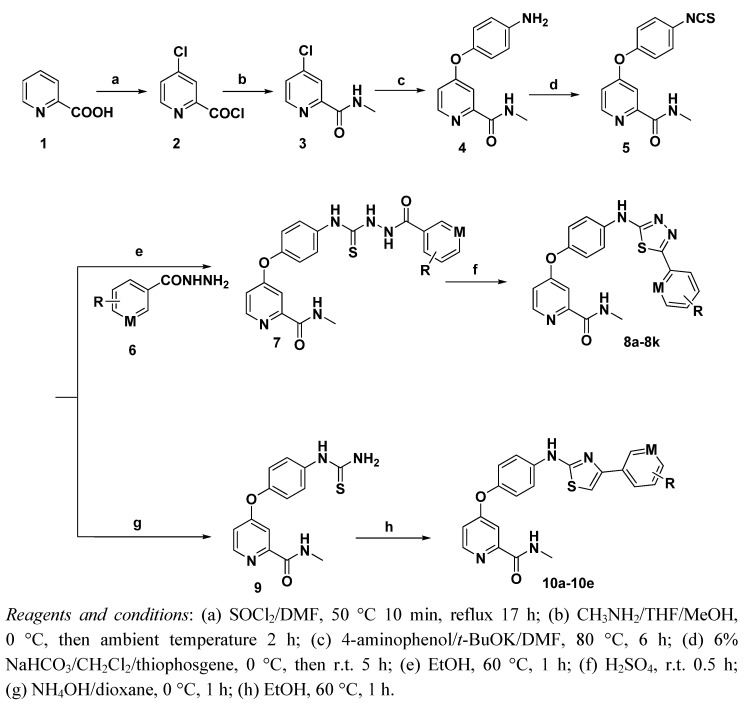
Synthetic routes for **8a–8k** and **10a–10e**.

The synthetic routes for target compounds were illustrated as outlined in Scheme 1. Chlorination of the commercially available picolinic acid in thionyl chloride afforded **2**, which was subsequently reacted with 2.0 M methylamine in THF to give the corresponding compound **3** as pale-yellow crystals.

Etherification of **3** with 4-aminophenol in the presence of potassium *tert*-butoxide led to the formation of intermediate **4**. Compound **5** was prepared by reacting intermediate 4 with thiophosgene in chloroform and sodium bicarbonate as intermediate for both series of the target compounds. Condensation of **5** and arylhydrazide **6** in ethanol furnished the key intermediates **7a–7k** in good yield. Self-cyclization of **7** in sulfuric acid gave the target compounds **8a–8k**. Treating **5** with ammonium hydroxide in dioxane at 0 °C yielded the primary thiourea **9**. Subsequently, heterocyclization of **9** with 2-bromo-1-arylethanoned in the presence of ethanol afforded the target compounds **10a–10e**.

### 2.2. Antiproliferative Activities

The antiproliferative activity of target compounds **8a–8k** and **10a–10e** was evaluated *in vitro* by MTT assay with sorafenib as reference drug on three human cancer cell lines, including the non-small cell lung cancer cell line A549, the non-small cell lung cancer cell line H460 and the human colorectal cancer cell line HT-29. The biological activity data was presented in Table 1. Some of the compounds exhibited enhanced antiproliferative activity in low micromolar range against one or more cell lines compared to sorafenib. Especially, the most promising compound **8e** inhibited the proliferation of A549, H460 and HT29 cell lines with IC_50_ values of 3.6, 1.7 and 3.0 μM, respectively.

**Table 1:** Cytotoxic activity of target compounds against A549, H460 and HT-29 cell lines *in vitro*. 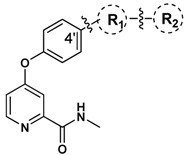

Compound	Substituent		IC_50 _(μM)		
	R_1_	R_2_	A549	H460	HT-29
**8a**	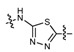	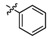	35.1	59.7	6.7
**8b**	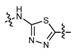	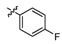	a NA	21.7	14.2
**8c**	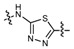	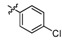	6.2	5.6	3.3
**8d**	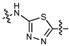	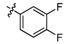	16.4	3.2	5.1
**8e**	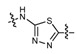	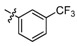	3.6	1.7	3.0
**8f**	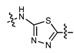	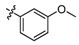	65.3	a NA	6.6
**8g**	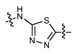	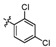	15.8	51.2	4.3
**8h**	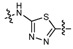	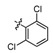	a NA	a NA	18.6
**8i**	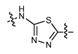	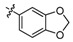	9.4	7.2	3.8
**8j**	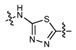	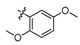	4.2	2.3	2.4
**8k**	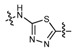	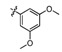	3.5	2.1	4.5
**10a**	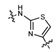	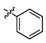	a NA	a NA	a NA
**10b**	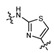	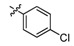	a NA	a NA	14.2
**10c**	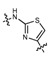	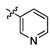	a NA	a NA	43.8
**10d**	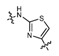	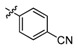	a NA	77.4	34.3
**10e**	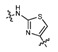	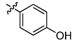	a NA	a NA	a NA
**Sorafenib**	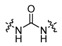	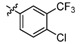	1.3	2.7	3.7

^a^ NA means not active.

As shown in Table 1, five compounds (**8c**, **8d**, **8e**, **8j** and **8k**) exhibited good cytotoxicity in low micromolar range against two or more cell lines. The pharmacological data suggested that compounds **8a–8k** displayed enhanced cytotoxic activity against HT-29 cell line as well as the prominent selectivity. In addition, introduction of the thiazol-2-ylamino backbone to the 4′-position of the *N-*methyl-4-phenoxypicolinamide (compounds **10a–10e**) resulted in diminished or even disappearance of the cytotoxicity. In contrast, most compounds with a 1,3,4-thiadiazol-2-ylamino group displayed better cytotoxicity than compounds with a thiazol-2-ylamino skeleton. It was speculated that the hydrogen bond building ability of R_1_ may influence the cytotoxity dramatically. Further studies focusing on investigating the influence of R_1 _on the cytotoxity are in progress in our laboratory and will be reported soon.

## 3. Experimental

### 3.1. General

All melting points were obtained on a Büchi Melting Point B-540 apparatus (Büchi Labortechnik, Flawil, Switzerland) and were uncorrected. Mass spectra (MS) were taken in ESI mode on Agilent 1100 LC-MS (Agilent, Palo Alto, CA, USA). NMR spectroscopy was performed using a 300 MHz Bruker ARX-300 spectrometer (Bruker Bioscience, Billerica, MA, USA) with DMSO-d_6_ as solvent and TMS as an internal standard. Column chromatography was run on silica gel (200–300 mesh) from Qingdao Ocean Chemicals (Qingdao, Shandong, China). Unless otherwise noted, all the reagents were obtained from commercially available sources and were used without further purification. 

### 3.2. Synthesis

#### 3.2.1. 4-Chloropicolinoyl chloride (**2**)

Anhydrous *N,N*-dimethylformamide (0.1 mL) was added to thionyl chloride (90 mL) at 50 °C under nitrogen. The solution was stirred at 50 °C for 10 min prior to portionwise addition of picolinic acid **1** (30 g, 0.244 mol) over 30 min. The initial green color went to orange and then to purple. The solution was heated to reflux, and vigorous SO_2_ evolution was observed. A yellow solid precipitated after 17 h. The mixture was then cooled to room temperature, diluted with toluene (200 mL), and concentrated under reduced pressure to about 70 mL. This process was repeated two additional times to give **2** as a brown oil which was used in the next step without further purification.

#### 3.2.2. 4-Chloro-*N*-methylpicolinamide (**3**)

4-Chloropicolinoyl chloride (**2**, 20.0 g, 113.7 mmol) was added portionwise to 2.0 M methylamine in tetrahydrofuran (350 mL) and methanol (70 mL) at 0 °C. The mixture was stirred at ambient temperature for 2 h, concentrated to near dryness, and dissolved in ethyl acetate (350 mL). The organic phase was washed with brine (350 mL), dried over sodium sulfate, and concentrated to provide **3** (16.0 g, 94.3 mmol, 83%) as a yellow, crystalline solid. m.p.: 41–42 °C. ^1^H-NMR δ: 8.87–8.85 (m, 1H, amide–***NH***), 8.61 (d, *J* = 5.1 Hz, 1H, pyridine–***6H***), 8.01 (d, *J* = 2.4 Hz, 1H, pyridine-***3H***), 7.75–7.73 (q, *J* = 2.4, 5.7 Hz, 1H, pyridine–***5H***), 2.82 (d, *J* = 5.1 Hz, 3H, ***CH_3_***); ESI-MS *m/z*: 171.3 (M+H)^+^.

#### 3.2.3. 4-(4-Aminophenoxy)-*N*-methylpicolinamide (**4**)

A solution of 4-aminophenol (9.6 g, 88.0 mmol) in dry *N*,*N*-dimethylformamide (150 mL) was treated with potassium *tert*-butoxide (10.29 g, 91.69 mmol), and the reddish-brown mixture was stirred at room temperature for 2 h. The contents were treated with 4-chloro-*N*-methylpicolinamide (**3**, 15.0 g, 87.9 mmol) and potassium carbonate (6.5 g, 47.0 mmol) and then heated to 80 °C under nitrogen for 6 h. The mixture was cooled to room temperature and poured into the mixture of ethyl acetate (500 mL) and brine (500 mL). The layers were separated, and the aqueous phase was back-extracted with ethyl acetate (300 mL). The combined organics were washed with brine (4 × 300 mL), dried over sodium sulfate, and concentrated to afford **4 **(17.1 g, 70.3 mmol, 80%) as a purple solid. ^1^H-NMR δ: 8.74–8.72 (m, 1H, amide–***NH***), 8.45 (d, *J* = 5.4 Hz, 1H, pyridine–***6H***), 7.34 (d, *J* = 3 Hz, 1H, pyridine–***3H***), 7.07–7.05 (q, *J* = 3, 5.4 Hz, 1H, pyridine–***5H***), 6.87 (d, *J* = 8.4 Hz, 2H, phenyl–***3H***,***5H***), 6.66 (d, *J* = 8.7 Hz, 2H, phenyl–***2H***, ***6H***), 2.78 (d, *J* = 4.8 Hz, 3H, ***CH_3_***); ESI-MS *m/z*: 244.0 (M+H)^+^.

#### 3.2.4. 4-(4-Isothiocyanatophenoxy)-*N*-methylpicolinamide (**5**)

To a stirred solution of 4-(4-aminophenoxy)-*N*-methylpicolinamide **4** (17.1 g, 70.3 mmol) in 1300 mL of 6% NaHCO_3_ solution was added 600 mL CH_2_Cl_2_. After 20 min of vigorous stirring at 0 °C, thiophosgene (4.1 mL, 70.3 mmol) was added dropwise. The reaction mixture was left under stirring for 5 h at room temperature, and the organic solvent was removed under reduced pressure and the crude residue was washed with cold ethanol to afford **5** (15.4 g, 54.1 mmol, 77%) as a brown powder. ^1^H-NMR δ: 8.79–8.78 (m, 1H, amide–***NH***), 8.54 (d, *J* = 6 Hz, 1H, pyridine–***6H***), 7.59 (d, *J* = 9.3 Hz, 2H, phenyl–***3H***,***5H***), 7.43(d, *J* = 3 Hz, 1H, pyridine–***3H***), 7.32 (d, *J* = 8.7 Hz, 2H, phenyl–***2H***,***6H***), 7.20–7.17 (q, *J* = 3, 6 Hz, 1H, pyridine–***5H***), 2.80 (d, *J* = 4.5 Hz, 3H); ESI-MS *m/z*: 286.2 (M+H)^+^.

#### 3.2.5. 4-(4-(2-Arylhydrazinecarbothioamido)phenoxy)-*N*-methylpicolinamides **7a–7k**

A mixture of arylhydrazide (27.0 mmol) **6** and 4-(4-isothiocyanatophenoxy)-*N*-methylpicolinamide (**5**, 27.0 mmol) in ethanol (60 mL) was placed in a flask and refluxed for 2 h. The mixture was allowed to cool to ambient temperture, and then the solvent was removed by reduced pressure distillation to furnish the key intermediates **7a–7k** which were pure enough to be used in the next step without further purification.

#### 3.2.6. General procedure for the preparation of compound **8a–8k**

4-(4-(2-arylhydrazinecarbothioamido)phenoxy)-*N*-methylpicolinamides **7a–7k** (0.26 mmol) were added portionwise to 3 mL concentrated sulfuric acid and stirred at room temperature for 30 min. Then crushed ice (10 g) was add to the mixture, followed by ammonia was used to neutralize the solution. The crude product precipitated was purified by chromatography on silica gel using 30:1 MeOH/CH_2_Cl_2_ as eluent.

*N-Methyl-4-(4-(5-phenyl-1,3,4-thiadiazol-2-ylamino)phenoxy)picolinamide* (**8a**). Yield: 76%. m.p.: 233–234 °C. MS [MH^−^] (*m/z*): 404.2 (M−1); ^1^H-NMR δ: 10.71 (s, 1H, –***NH***), 8.77–8.75 (m, 1H, amide–***NH***), 8.52 (d, *J* = 6.0 Hz, 1H, pyridine–***6H***), 7.88–7.86 (m, 2H, phenyl–***3H***,***5H***), 7.81 (d, *J* = 9.0 Hz, 2H, phenyl–***3H***,***5H***), 7.52–7.51 (m, 3H, phenyl–***2H***,***4H***,***6H***), 7.40 (d, *J* = 3.0 Hz, 1H, pyridine–***3H***), 7.28 (d, *J* = 9.0 Hz, 2H, phenyl–***2H***,***6H***), 7.17 (q, *J* = 3.0, 6.0 Hz, 1H, pyridine–***5H***), 2.79 (d, *J* = 6.0 Hz, 3H, ***CH_3_***); ^13^C-NMR δ: 171.5 (C), 164.3 (C), 159.7 (C), 151.0 (C), 146.6 (C), 141.2 (CH), 138.9 (C), 134.4 (C), 130.2 (C), 127.8 (CH), 127.8 (CH), 126.3 (CH), 126.3 (CH), 125.8 (CH), 123.5 (CH), 123.5 (CH), 119.8 (CH), 119.8 (CH), 110.1 (CH), 107.3 (CH), 26.1 (CH_3_); Anal. Calcd for C_2__1_H_17_N_5_O_2_S (%): C, 64.08; H, 4.38; N, 15.64; found C 63.01, H 4.05, N 15.21.

*N-Methyl-4-(4-(5-(4-fluorophenyl)-1,3,4-thiadiazol-2-ylamino)phenoxy)picolinamide* (**8b**). Yield: 52%. m.p.: 219–220 °C. MS [MH^−^] (*m/z*): 422.2 (M−1); ^1^H-NMR δ: 10.77 (s, 1H, –***NH***), 8.77–8.75 (br, 1H, amide–***NH***), 8.51 (d, *J* = 6.0 Hz, 1H, pyridine–***6H***), 7.95–7.90 (m, 2H, phenyl–***3H***,***5H***), 7.82 (d, *J* = 9.0 Hz, 2H, phenyl–***3H***,***5H***), 7.40–7.33 (m, 3H, phenyl–***2H***,***6H***, pyridine-***3H***), 7.26 (d, *J* = 9.0 Hz, 2H, phenyl-***2H***,***6H***), 7.16–7.14 (m, 1H, pyridine–***5H***), 2.79 (d, *J* = 6.0 Hz, 3H, ***CH_3_***); ^13^C-NMR δ: 173.3 (C), 163.8 (C), 162.5 (C), 151.2 (C), 148.8 (C), 143.5 (CH), 140.6 (C), 137.1 (C), 130.2 (C), 129.7 (CH), 127.3 (CH), 127.3 (CH), 125.6 (CH), 125.6 (CH), 123.5 (CH), 123.5 (CH), 119.8 (CH), 119.8 (CH), 111.9 (CH), 110.7 (CH), 26.1 (CH_3_); Anal. Calcd for C_2__1_H_1__6_ FN_5_O_2_S (%): C, 59.85; H, 3.83; N, 16.62; found C 60.03, H 3.99, N 16.83.

*N-Methyl-4-(4-(5-(4-chlorophenyl)-1,3,4-thiadiazol-2-ylamino)phenoxy)picolinamide* (**8c**). Methylene Yield: 69%. m.p.: 245–246 °C. MS [MH^−^] (*m/z*): 439.2 (M−1); ^1^H-NMR δ: 10.77 (s, 1H, –***NH***), 8.78–8.74 (br, 1H, amide–***NH***), 8.50 (d, *J* = 6.0 Hz, 1H, pyridine–***6H***), 7.89 (d, *J* = 9.0 Hz, 2H, phenyl–***3H***,***5H***), 7.80 (d, *J* = 9.0 Hz, 2H, phenyl–***3H***,***5H***), 7.58 (d, 2H, *J* = 9.0 Hz, phenyl–***2H***,***6H***), 7.39 (s, 1H, pyridine–***3H***), 7.25 (d, *J* = 9.0 Hz, 2H, phenyl–***2H***,***6H***), 7.15–7.14 (br, 1H, pyridine–***5H***), 2.78 (d, *J* = 6.0 Hz, 3H, ***CH_3_***); ^13^C-NMR δ: 173.5 (C), 164.3 (C), 160.6 (C), 152.1(C), 150.8(C), 144.3 (CH), 141.9 (C), 136.4 (C), 130.9 (C), 126.3 (CH), 126.3 (CH), 125.7 (CH), 125.7 (CH), 123.8 (CH), 121.3 (CH), 121.3 (CH), 119.6 (CH), 119.6 (CH), 109.9 (CH), 107.3 (CH), 26.1 (CH_3_); Anal. Calcd for C_2__1_H_1__6_ ClN_5_O_2_S (%): C, 57.60; H, 3.68; N, 15.99; found C 57.82, H 3.75, N 16.18.

*N-Methyl-4-(4-(5-(3,4-difluorophenyl)-1,3,4-thiadiazol-2-ylamino)phenoxy)picolinamide* (**8d**). Yield: 40%. m.p.: 225–226 °C. MS [MH^−^] (*m/z*): 440.7 (M−1); ^1^H-NMR δ: 10.83 (s, 1H, –***NH***), 8.78–8.75 (br, 1H, amide–***NH***), 8.51 (d, *J* = 6.0 Hz, 1H, pyridine–***6H***), 7.99–7.93 (m, 1H, phenyl–***5H***), 7.81–7.71 (m, 3H, phenyl–***3H***,***5H***, phenyl–***2H***), 7.63–7.54 (q, *J* = 6.0, 9.0 Hz, 1H, phenyl–***6H***), 7.39 (s, 1H, pyridine–***3H***), 7.26 (d, *J* = 9.0 Hz, 2H, phenyl–***2H***,***6H***), 7.14 (q, *J* = 3.0, 6.0 Hz, 1H, pyridine–***5H***), 2.79 (d, *J* = 6.0 Hz, 3H, ***CH_3_***); ^13^C-NMR δ: 171.9 (C), 164.8 (C), 161.1 (C), 151.4(C), 150.6(C), 148.4 (C), 147.3 (C), 145.9(CH), 141.6 (C), 135.8 (C), 130.3 (C), 128.9 (CH), 124.3 (CH), 124.3 (CH), 121.5 (CH), 121.5 (CH), 119.6 (CH), 119.6 (CH), 111.4 (CH), 109.1 (CH), 26.1 (CH_3_); Anal. Calcd for C_2__1_H_1__5_F_2_N_5_O_2_S (%): C, 57.40; H, 3.44; N, 15.94; found C 58.21, H 3.63, N 16.27.

*N-Methyl-4-(4-(5-(3-(trifluoromethyl)phenyl)-1,3,4-thiadiazol-2-ylamino)phenoxy)picolinamide* (**8e**). Yield: 55%. m.p.: 242–243 °C. MS [MH^+^] (*m/z*): 472.6 (M+1); ^1^H-NMR δ: 10.85 (s, 1H, –***NH***), 8.76–8.75 (br, 1H, amide–***NH***), 8.51 (d, *J* = 6.0 Hz, 1H, pyridine–***6H***), 8.16 (br, 2H, pyridine–***2H***,***4H***), 7.87–7.76 (m, 4H, phenyl–***3H***,***5H***, phenyl–***5H***,***6H***), 7.40 (s, 1H, pyridine–***3H***), 7.27 (d, *J* = 9.0 Hz, 2H, phenyl–***2H***,***6H***), 7.16–7.13 (br, 1H, pyridine–***5H***), 2.78 (d, *J* = 6.0 Hz, 3H, ***CH_3_***); ^13^C-NMR δ: 173.3 (C), 165.2 (C), 160.5 (C), 154.6(C), 149.9(C), 144.2 (CH), 141.3 (C), 133.5(C), 133.1 (CH), 132.9 (C), 130.5 (C) 129.5 (CH), 126.5 (CH), 125.1 (CH), 124.4 (CH), 121.6 (CH), 121.6 (CH), 119.6 (CH), 119.6 (CH), 112.7 (CH), 108.9 (CH), 26.2 (CH_3_); Anal. Calcd for C_2__2_H_1__6_F_3_N_5_O_2_S (%): C, 56.05; H, 3.42; N, 14.85; found C 56.21, H 3.69, N 15.03.

*N-Methyl-4-(4-(5-(3-methoxyphenyl)-1,3,4-thiadiazol-2-ylamino)phenoxy)picolinamide* (**8f**). Yield: 82%. m.p.: 237–238 °C. MS [MH^−^] (*m/z*): 434.2 (M−1); ^1^H-NMR δ: 10.61 (s, 1H, –***NH***), 8.77–8.75 (m, 1H, amide–***NH***), 8.51 (d, *J* = 6.0 Hz, 1H, pyridine–***6H***), 7.82 (d, *J* = 9.0 Hz, 2H, phenyl–***3H***,***5H***), 7.74(d, *J* = 6.0 Hz, *J* = 9.0 Hz, 2H, phenyl–***4H***,***6H***), 7.40 (d, *J* = 3.0 Hz, 1H, pyridine–***3H***),7.25 (d, *J* = 9.0 Hz, 2H, phenyl–***2H***,***6H***), 7.16 (q, *J* = 3.0, 6.0 Hz, 1H, pyridine–***5H***), 7.00 (s, 2H, phenyl–***2H***,***5H***), 3.81 (s, 3H, ***CH_3_***), 2.79 (d, *J* = 6.0 Hz, 3H, ***CH_3_***); ^13^C-NMR δ: 173.5 (C), 162.8 (C), 160.3 (C), 160.0 (C), 153.3 (C), 152.6 (CH), 145.6 (CH), 141.7 (C), 135.8 (C), 133.7 (C), 131.9 (CH) 122.2 (CH), 120.9 (CH), 120.9 (CH), 118.9 (CH), 118.9 (CH), 113.6 (CH), 113.1 (CH), 111.3 (CH), 108.6 (CH), 55.6 (CH_3_), 26.1 (CH_3_); Anal. Calcd for C_2__2_H_1__9_N_5_O_3_S (%): C, 60.96; H, 4.42; N, 16.16; found C 60.57, H 4.49, N 16.13.

*N-Methyl-4-(4-(5-(2,4-dichlorophenyl)-1,3,4-thiadiazol-2-ylamino)phenoxy)picolinamide* (**8g**). Yield: 68%. m.p.: 251–252 °C. MS [MH^+^] (*m/z*): 473.0 (M+1); ^1^H-NMR δ: 10.95 (s, 1H, –***NH***), 8.77–8.75 (m, 1H, amide–***NH***), 8.51 (d, *J* = 6.0 Hz, 1H, pyridine–***6H***), 8.11 (d, *J* = 9.0 Hz, 1H, phenyl–***3H***), 7.86–7.81 (m, 3H, phenyl–***3H***,***5H***, phenyl–***5H***), 7.62 (q, *J* = 3.0, 9.0 Hz, 2H, phenyl–***6H***), 7.39 (d, 1H, *J* = 3.0 Hz, pyridine–***3H***), 7.26 (d, *J* = 9.0 Hz, 2H, phenyl–***2H***,***6H***), 7.16 (q, *J* = 3.0, 6.0 Hz, 1H, pyridine–***5H***), 2.79 (d, *J* = 6.0 Hz, 3H, ***CH_3_***); ^13^C-NMR δ: 173.7 (C), 163.6 (C), 160.9 (C), 151.7 (C), 151.0 (C), 146.2 (CH), 142.4 (C), 136.4 (C), 135.7 (C), 135.0 (C), 133.6 (C), 130.9 (CH), 130.3 (CH) 127.4 (CH), 120.5 (CH), 120.5 (CH), 119.1 (CH), 119.1 (CH), 113.9 (CH), 108.6 (CH), 26.1 (CH_3_); Anal. Calcd for C_2__1_H_1__5_Cl_2_N_5_O_2_S (%): C, 53.40; H, 3.20; N, 14.83; found C 54.07, H 3.31, N 15.01.

*N*-methyl-4-(4-(5-(2,6-dichlorophenyl)-1,3,4-thiadiazol-2-ylamino)phenoxy)picolinamide (**8h**). Yield: 72%. m.p.: 261–262 °C. MS [MH^−^] (*m/z*): 473.8 (M−1); ^1^H-NMR δ: 10.90 (s, 1H, –***NH***), 8.77–8.75 (m, 1H, amide–***NH***), 8.51 (d, *J* = 6.0 Hz, 1H, pyridine–***6H***), 7.84 (d, *J* = 9.0 Hz, 2H, phenyl–***3H***,***5H***), 7.69 (d, *J* = 9.0 Hz, 2H, phenyl–***3H***,***5H***), 7.62–7.57 (m, 1H, phenyl–***4H***), 7.41 (d, *J* = 3.0 Hz, 1H, pyridine–***3H***), 7.27 (d, *J* = 9.0 Hz, 2H, phenyl–***2H***, ***6H***), 7.16 (q, *J* = 3.0, 6.0 Hz, 1H, pyridine–***5H***), 2.80 (d, *J* = 6.0 Hz, 3H, ***CH_3_***); ^13^C-NMR δ: 173.5 (C), 162.9 (C), 160.3 (C), 151.8 (C), 150.6 (C), 145.5 (CH), 141.7 (C), 138.0 (C), 136.3 (C), 134.7 (C), 134.7 (C), 131.5 (CH), 127.4 (CH), 127.4 (CH) 121.3 (CH), 121.3 (CH), 120.1 (CH), 120.1 (CH), 113.9 (CH), 108.6 (CH), 26.1 (CH_3_); Anal. Calcd for C_2__1_H_1__5_Cl_2_N_5_O_2_S (%): C, 53.40; H, 3.20; N, 14.83; found C 53.67, H 3.34, N 14.89.

N*-Methyl-4-(4-(5-(benzo[d][1,3]dioxol-5-yl)-1,3,4-thiadiazol-2-ylamino)phenoxy)picolinamide* (**8i**). Yield: 47%. m.p.: 243–244 °C. MS [MH^+^] (*m/z*): 448.2 (M+1); ^1^H-NMR δ: 10.65 (s, 1H, -***NH***), 8.77–8.76 (br, 1H, amide–***NH***), 8.51 (d, *J* = 6.0 Hz, 1H, pyridine–***6H***), 7.79 (d, *J* = 9.0 Hz, 2H, phenyl–***3H***,***5H***), 7.43–7.33 (m, 3H, phenyl–***2H***, ***6H***, pyridine–***3H***), 7.25 (d, *J* = 9.0 Hz, 2H, phenyl–***2H***,***6H***), 7.05 (d, 1H, *J* = 9.0 Hz, phenyl–***5H***), 6.77–6.71 (m, 1H, pyridine–***5H***), 2.79 (d, *J* = 6.0 Hz, 3H, ***CH_3_***); ^13^C-NMR δ: 174.1 (C), 164.8 (C), 161.0 (C), 152.7 (C), 151.0 (C), 146.6 (C), 146.2 (CH), 145.2 (C), 142.4 (C), 136.4 (C), 123.1 (C), 122.5 (CH), 121.7 (CH), 121.7 (CH), 120.8 (CH), 120.0 (CH), 120.0 (CH), 115.3 (CH), 113.9 (CH), 109.6 (CH), 101.5 (CH_2_), 26.1 (CH_3_); Anal. Calcd for C_2__2_H_1__7_N_5_O_4_S (%): C, 59.05; H, 3.83; N, 15.65; found C 58.73, H 3.81, N 15.59.

*N-Methyl-4-(4-(5-(2,5-dimethoxyphenyl)-1,3,4-thiadiazol-2-ylamino)phenoxy)picolinamide* (**8j**). Yield: 63%. m.p.: 267–268 °C. MS [MH^−^] (*m/z*): 464.7 (M−1); ^1^H-NMR δ: 10.57 (s, 1H, -***NH***), 8.76–8.75 (br, 1H, amide–***NH***), 8.51 (d, *J* = 6.0 Hz, 1H, pyridine–***6H***), 7.82 (d, *J* = 9.0 Hz, 2H, phenyl–***3H***,***5H***), 7.74 (d, *J* = 3.0 Hz, 1H, phenyl–***6H***), 7.40 (s, 1H, pyridine–***3H***), 7.25–7.18 (m, 3H, phenyl–***2H***,***6H***, phenyl–***2H***), 7.16–7.14 (br, 1H, pyridine–***5H***), 7.10 (q, *J* = 3.0 Hz, 6.0 Hz, 1H, pyridine–***5H***), 2.93 (s, 3H, ***CH_3_***), 3.79 (s, 3H, ***CH_3_***), 2.79 (d, *J* = 6.0 Hz, 3H, ***CH_3_***); ^13^C-NMR δ: 173.8 (C), 164.2 (C), 160.5 (C), 151.9 (C), 151.8 (C), 150.3 (C), 148.7 (C), 144.1 (CH), 141.3 (C), 135.8 (C), 121.1 (C), 121.0 (CH), 121.0 (CH), 118.9 (CH), 118.9 (CH), 115.3 (CH), 113.7 (CH), 112.3 (CH), 111.9 (CH), 109.6 (CH), 56.1 (CH_3_), 55.8 (CH_3_), 26.1 (CH_3_); Anal. Calcd for C_2__3_H_21_N_5_O_4_S (%): C, 59.60; H, 4.57; N, 15.11; found C 59.28, H 4.45, N 14.89.

*N-Methyl-4-(4-(5-(3,5-dimethoxyphenyl)-1,3,4-thiadiazol-2-ylamino)phenoxy) picolinamide* (**8k**). Yield: 72%. m.p.: 250–251 °C. MS [MH^+^] (*m/z*): 464.3 (M+1); ^1^H-NMR δ: 10.79 (s, 1H, -***NH***), 8.75–8.74 (br, 1H, amide–***NH***), 8.50 (d, *J* = 6.0 Hz, 1H, pyridine–***6H***), 7.80 (d, *J* = 9.0 Hz, 2H, phenyl–***3H***,***5H***), 7.39 (s, 1H, pyridine–***3H***), 7.24 (d, 2H, *J* = 9.0 Hz, phenyl–***2H***, ***6H***), 7.15–7.13 (br, 1H, pyridine–***5H***), 6.97 (s, 2H, phenyl–***2H***,***6H***), 6.62 (s, 1H, phenyl–***4H***), 3.81 (s, 3H, ***CH_3_***), 2.78 (d, *J* = 6.0 Hz, 3H, ***CH_3_***); ^13^C-NMR δ: 173.5 (C), 164.2 (C), 160.6 (C), 158.9 (C), 158.9 (C), 151.9 (C), 151.0 (C), 145.3 (CH), 142.8 (C), 135.9 (CH), 134.9 (C), 121.0 (CH), 121.0 (CH), 118.9 (CH), 118.9 (CH), 113.8 (CH), 108.6 (CH), 102.9 (CH), 102.9 (CH), 100.1 (CH), 55.7 (CH_3_) , 55.3 (CH_3_) , 26.1 (CH_3_). Anal. Calcd for C_2__3_H_21_N_5_O_4_S (%): C, 59.60; H, 4.57; N, 15.11; found C 60.18, H 4.73, N 15.26.

#### 3.2.7. *N*-methyl-4-(4-thioureidophenoxy)picolinamide (**9**)

To a mixture of ammonium hydroxide (10 mL) and dioxane (60 mL) was added **5 **(15.4 g, 54.1 mmol), and keep the temperature at 0 °C for a period of 1 h until most of the product participated from the reaction mixture. Crude product was obtained after filtration. The solid obtained was recrystalized in ethanol to yield an off white solid **9** (13.1 g, 43.2 mmol, 80%) after filtration. m.p.: 72–73 °C. ^1^H-NMR (DMSO) δ: 9.77 (br, 1H), 8.79–8.78 (m, 1H), 8.52 (d, *J* = 6.0 Hz, 1H), 7.54 (d, *J* = 9.0 Hz, 2H), 7.42 (d, *J* = 2.7 Hz, 1H), 7.20–7.14 (m, 3H), 2.79 (d, *J* = 4.8 Hz, 3H); ESI-MS *m/z*: 303.1 (M+H)^+^.

#### 3.2.8. General procedure for preparation of compound (**10a–10e**)

A mixture of *N*-methyl-4-(4-thioureidophenoxy)picolinamide (**9**, 0.16 g, 0.5 mmol) and 2-bromo-1-arylethanone (0.5 mmol) was refluxed in anhydrous ethanol (5 mL) for 1h. Then mixture was allowed to precipitate enough crude product at room temperature for 1 h and compound **10a–10e** was obtained after filtration and recrystallation in ethanol.

*N-Methyl-4-(4-(4-phenylthiazol-2-ylamino)phenoxy)picolinamide* (**10a**). 2-bromo-1-phenylethanone. Yield: 79%. m.p.: 271–272 °C. MS [MH^+^] (*m/z*): 403.2 (M+1); ^1^H-NMR δ: 10.52 (s, 1H, –***NH***), 9.03–9.01 (m, 2H, amide–***NH***, pyridine–***6H***), 8.57–8.55 (m, 1H, pyridine–***5H***), 7.92–7.89 (m, 3H, thiazole–***H***, phenyl–***3H***, ***5H***), 7.81 (d, *J* = 3.0 Hz, 1H, pyridine–***3H***), 7.47–7.36 (m, *4*H, phenyl–***2H***, ***6H***, phenyl–***2H***, ***6H***), 7.29–7.22 (m, 3H, phenyl–***3H***, ***4H***, ***5H***), 2.79 (d, *J* = 6.0 Hz, 3H, ***CH_3_***); ^13^C-NMR δ: 163.9 (C), 160.2 (C), 160.0 (C), 150.3 (C), 149.1 (C), 145.6 (CH), 141.5 (C), 136.0 (C), 133.9 (C), 129.0 (CH), 129.0 (CH), 128.1 (CH), 127.1 (CH), 127.1 (CH), 121.2 (CH), 121.2 (CH), 119.3 (CH), 119.3 (CH), 113.5 (CH), 109.2 (CH), 105.0 (CH), 26.1 (CH_3_); Anal. Calcd for C_2__2_H_18_N_4_O_2_S (%): C, 65.65; H, 4.51; N, 13.92; found C 65.88, H 4.39, N 14.02.

*N*-*Methyl-4-(4-(4-(4-chlorophenyl)thiazol-2-ylamino)phenoxy)picolinamide* (**10b**). 2-bromo-1-(4-chlorophenyl)ethanone. Yield: 88%. m.p.: 278–279 °C. MS [MH^+^] (*m/z*): 437.3 (M+1); ^1^H-NMR δ: 10.67 (s, 1H, –***NH***), 8.99–8.92 (br, 1H, amide–***NH***), 8.55–8.53 (m, 1H, pyridine–***6H***), 7.94 (d, *J* = 9.0 Hz, 2H, phenyl–***3H***,***5H***), 7.88 (d, 1H, *J* = 3.0 Hz, pyridine–***3H***), 7.78 (d, 2H, *J* = 9.0 Hz, phenyl–***3H***,***5H***), 7.61–7.58 (br, 1H, pyridine–***5H***), 7.53 (d, 2H, *J* = 9.0 Hz, phenyl–***2H***, ***6H***), 7.24–7.21 (m, 3H, phenyl–***2H***,***6H***, thiazole–***H***), 2.79 (d, *J* = 6.0 Hz, 3H, ***CH_3_***); ^13^C-NMR δ: 163.8 (C), 161.0 (C), 160.3 (C), 150.2 (C), 148.9 (C), 146.2 (CH), 142.1 (C), 135.4 (C), 133.8 (C), 131.0 (C), 129.3 (CH), 129.3 (CH), 128.5 (CH), 128.5 (CH), 121.2 (CH), 121.2 (CH), 119.6 (CH), 119.6 (CH), 113.2 (CH), 109.8 (CH), 105.1 (CH), 26.3 (CH_3_); Anal. Calcd for C_2__2_H_17_ ClN_4_O_2_S (%): C, 60.48; H, 3.92; N, 12.82; found C 60.73, H 3.97, N 13.03.

*N*-*Methyl-4-(4-(4-(pyridin-3-yl)thiazol-2-ylamino)phenoxy)picolinamide* (**10c**). 2-bromo-1-(pyridin-3-yl)ethanone. Yield: 82%. m.p.: 266–267 °C. MS [MH^+^] (*m/z*): 404.2 (M+1); ^1^H-NMR δ: 10.72 (s, 1H, –***NH***), 9.37(s, 1H, pyridine–***3H***), 9.02–9.01 (br, 1H, amide–***NH***), 8.85–8.82 (m, 2H, pyridine–***6H***, pyridine–***6H***), 8.53 (d, *J* = 3.0 Hz, 1H, pyridine–***4H***), 8.11 (q, *J* = 3.0, 6.0 Hz, 1H, pyridine–***5H***), 7.93–7.89 (m, 3H, phenyl–***3H***,***5H***, pyridine–***3H***), 7.47 (s, 1H, thiazole–***H***), 7.23–7.19 (m, 3H, phenyl–***2H***,***6H***, pyridine–***5H***), 2.78 (d, *J* = 6.0 Hz, 3H, ***CH_3_***); ^13^C-NMR δ: 163.4 (C), 160.0 (C), 158.2 (C), 149.9 (C), 146.7 (CH), 147.3 (CH), 146.1 (CH), 142.0 (C), 136.1 (C), 135.3 (C), 133.8 (CH), 133.8 (C), 124.0 (CH), 121.1 (CH), 121.1 (CH), 120.1 (CH), 120.1 (CH), 113.5 (CH), 109.6 (CH), 109.6 (CH), 26.3 (CH_3_); Anal. Calcd for C_2__1_H_17_ N_5_O_2_S (%): C, 62.52; H, 4.25; N, 17.36; found C 62.84, H 4.47, N 17.39.

*N*-*Methyl-4-(4-(4-(4-cyanophenyl)thiazol-2-ylamino)phenoxy)picolinamide* (**10d**). 4-(2-bromoacetyl)benzonitrile. Yield: 75%. m.p.: 281–282 °C. MS [MH^+^] (*m/z*): 428.6 (M+1); ^1^H-NMR δ: 10.56 (s, 1H, –***NH***), 8.88–8.87 (br, 1H, amide–***NH***), 8.53–8.51 (m, 1H, pyridine–***6H***), 8.12–8.09 (m, 2H, phenyl–***3H***,***5H***), 7.94–7.76 (m, 4H, phenyl–***2H***,***3H***,***5H***,***6H***), 7.67 (s, 1H, thiazole–***H***), 7.52–7.49 (m, 1H, pyridine–***3H***), 7.52–7.49 (m, 1H, phenyl–***2H***,***6H***, pyridine–***5H***), 2.79 (d, *J* = 6.0 Hz, 3H, ***CH_3_***); ^13^C-NMR δ: 164.0 (C), 161.1 (C), 160.5 (C), 151.2 (C), 149.7 (C), 146.5 (CH), 142.3 (C), 136.9 (C), 136.2 (C), 132.1 (CH), 132.1 (CH), 126.0 (CH), 126.0 (CH), 121.3 (CH), 121.3 (CH), 120.0 (CH), 120.0 (CH), 118.5 (CH), 113.7 (CH), 112.8 (CH), 109.3 (CH), 105.2 (CH), 26.3 (CH_3_); Anal. Calcd for C_2__3_H_17_ N_5_O_2_S (%): C, 64.62; H, 4.01; N, 16.38; found C 64.48, H 4.06, N 16.41.

*N*-*Methyl-4-(4-(4-(4-hydroxyphenyl)thiazol-2-ylamino)phenoxy)picolinamide* (**10e**). 2-bromo-1-(4-hydroxyphenyl)ethanone. Yield: 79%. m.p.: 275–276 °C. MS [MH^+^] (*m/z*): 417.3 (M+1); ^1^H-NMR δ: 10.39 (s, 1H, –***NH***), 8.91–8.85 (br, 1H, amide–***NH***), 8.53 (d, *J* = 3.0 Hz, 1H, pyridine–***6H***), 7.88 (m, 2H, phenyl–***3H***,***5H***), 7.88 (m, 2H, phenyl–***2H***,***6H***), 7.53–7.49 (m, 1H, pyridine–***3H***), 7.23–7.21 (m, 3H, phenyl–***2H***,***6H***, pyridine–***5H***), 7.07 (s, 1H, thiazole–***H***), 6.79 (m, 1H, phenyl–***3H***,***5H***), 2.79 (d, *J* = 6.0 Hz, 3H, ***CH_3_***); ^13^C-NMR δ: 163.9 (C), 160.1 (C), 159.3 (C), 157.5 (C), 150.0 (C), 148.9 (C), 147.3 (CH), 141.6 (C), 134.9 (C), 128.2 (CH), 128.2 (CH), 125.1 (C), 121.4 (CH), 121.4 (CH), 120.8 (CH), 120.8 (CH), 116.1 (CH), 116.1 (CH), 113.3 (CH), 109.2 (CH), 103.9 (CH), 26.3 (CH_3_); Anal. Calcd for C_2__2_H_18_ N_4_O_3_S (%): C, 63.14; H, 4.34; N, 13.39; found C 63.39, H 4.42, N 13.52.

### 3.3. Pharmacology

The cytotoxic activities of compounds **8a–8k** and **10a–10e** were evaluated on the A549, H460 and HT29 cell lines by the standard MTT assay. The cancer cell lines were cultured in minimum essential medium (MEM) supplemented with 10% fetal bovine serum (FBS). The cells were maintained at 37 °C in a moisture-saturated atmosphere containing 5% CO_2_. The compounds were used at concentrations ranging from 0.16 to 100 μg/mL, and sorafenib at the same concentrations was introduced as positive control. The assessment of antiproliferative activity was expressed as concentration inhibiting 50% of cancer cell growth (IC_50_). Approximately 4 × 10^3^ cells, suspended in MEM medium, were plated onto each well of a 96-well plate and plates were incubated in 5% CO_2_ at 37 °C for 24 h before treatment with the compounds to allow attachment to the wall of the plate. The test compounds **8a–8k** and **10a–10e** at indicated final concentrations were added to the culture medium and the cell cultures were continued for 72 h. Fresh MTT was added to each well at a terminal concentration of 5 μg/mL and incubated with cells at 37 °C for 4 h. The formazan crystals were dissolved in 100 μL DMSO each well, and the absorbency at 492 nm (for absorbance of MTT formazan) and 630 nm (for the reference wavelength) was measured with the ELISA reader. All of the compounds were tested twice in each cell line. The results expressed as IC_50_ (inhibitory concentration 50%) were the averages of three determinations and calculated by using the Bacus Laboratories Incorporated Slide Scanner (Bliss) software.

## 4. Conclusions

In this paper, sixteen novel *N-*methyl-4-phenoxypicolinamidederivatives bearing thiadiazole or thiazole backbones were synthesized and evaluated for their *in vitro* cytotoxic activity against A549, H460 and HT29 cell lines. Among all of these derivatives synthesized, the most promising compound **8e**, exhibited more potent cytotoxicity on H460 and HT-29 cell lines than the reference drug sorafenib with IC_50_ values of 1.7 and 3.0 μM, respectively.
